# Toward Self-Measures in Cochlear Implants: Daily and “Homemade” Impedance Assessment

**DOI:** 10.3389/fdgth.2020.582562

**Published:** 2020-11-09

**Authors:** Matias Parreño, Federico A. Di Lella, Florencia Fernandez, Carlos M. Boccio, Sebastian A. Ausili

**Affiliations:** ^1^Department of Otolaryngology, Hospital Italiano, Buenos Aires, Argentina; ^2^Department of Otolaryngology, University of Miami, Coral Gables, FL, United States

**Keywords:** cochlear implants, telehealth, objective measurements, electrical impedance, self-measures

## Abstract

**Introduction:** Cochlear implant (CI) impedance reflects the status of the electro neural interface, potentially acting as a biomarker for inner ear injury. Most impedance shifts are diagnosed retrospectively because they are only measured in clinical appointments, with unknown behavior between visits. Here we study the application and discuss the benefits of daily and remote impedance measures with software specifically designed for this purpose.

**Methods:** We designed software to perform CI impedance measurements without the intervention of health personnel. Ten patients were recruited to self-measure impedance for 30 days at home, between CI surgery and activation. Data were transferred to a secured online server allowing remote monitoring.

**Results:** Most subjects successfully performed measurements at home without supervision. Only a subset of measurements was missed due to lack of patient engagement. Data were successfully and securely transferred to the online server. No adverse events, pain, or discomfort was reported by participants.

**Discussion:** This work overviews a flexible and highly configurable platform for self-measurement CI impedance. This novel approach simplifies the CI standard of care by reducing the number of clinical visits and by proving useful and constant information to CI clinicians.

## Introduction

Cochlear implants are the most successful sensory prosthetic device in medicine. Research has demonstrated that CIs typically provide significant improvement in speech recognition for persons with severe to profound hearing loss (1, 2). Like all neural prostheses, the interface between electrodes and neural tissue is a critical aspect for adequate functioning (3). The measurement of intracochlear electrode impedance provides an indication of the status of the electrode–tissue interface, which may give important information for the clinician providing CI management (4). Normally, this is a quick (i.e., 1–2 min) and safe procedure, because it involves the use of subthreshold- or near-threshold level stimulation (5, 6). During the postoperative period, impedance measurement is routinely performed for CI-programming guidance, detecting device failures, and extrusion of electrode contacts. Moreover, this value is a biomarker for inner ear injury (i.e., fibrosis and ossification) that may help predict residual hearing loss or vertigo events (7, 8).

While electrode impedance measurement provides essential information regarding the CI and cochlear status, it is only performed in the patient's clinical appointments, and not much is known on the behavior of this parameter between visits (7–9). Thus, most impedance variations are diagnosed retrospectively when little can be done to correlate them with clinical presentation or to start pharmacological treatment. More frequent monitoring of CI impedance with available methods is not feasible, but currently the use of telemedicine can be used to improve clinical practice by performing constant monitoring of electrode impedance values.

Telemedicine made its way into the cochlear implant clinic in the last 10 years with advances in connectivity. This progress was supported with the development of remote-access applications and new telecommunication systems (10, 11). Audiologists started performing remote fitting and monitoring of implanted patients, allowing medical care while keeping patients at their homes with no significant differences regarding standard programming sessions (10–18).

Here, we study the application and discuss the benefits of assessing daily and remote impedance measures with a software specifically designed for CIs. Patients measured themselves at home for 30 days, and data were automatically uploaded to an encrypted cloud database. The procedure did not require supervision of any clinicians and was easily performed in all patients. During the 30 days over which a recipient's electrode impedance values were measured in this study, the researchers could retrieve this data at any time, which enables true remote monitoring of CI status and cochlear health.

## Methods

### Hardware

**Figure 1:**
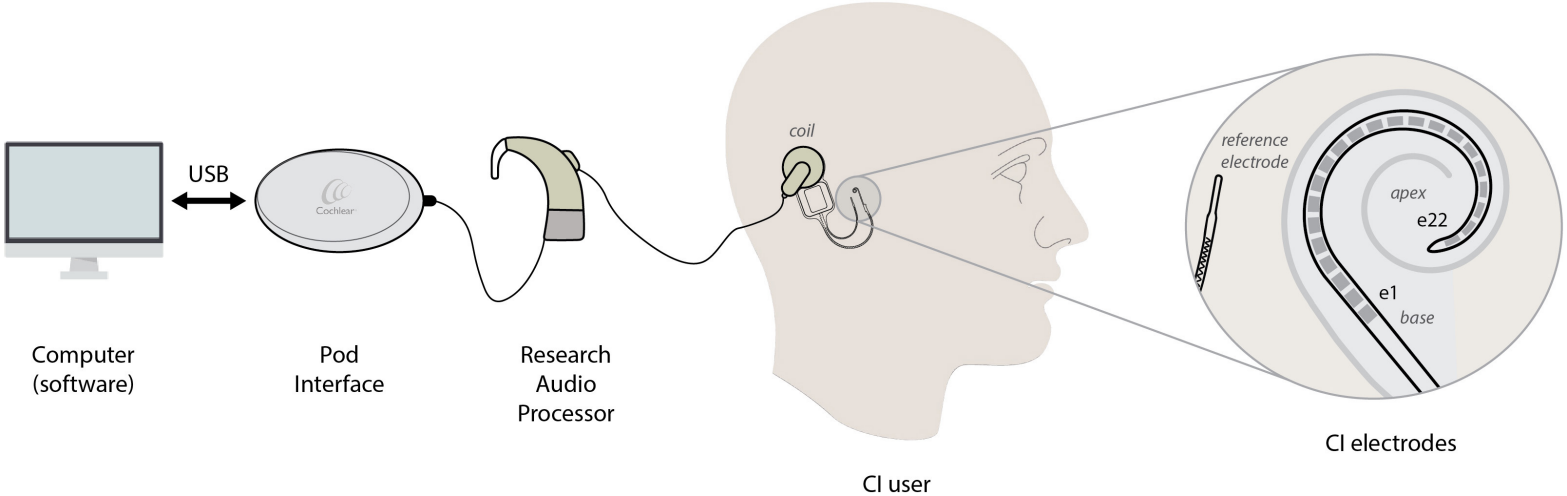
Hardware setup for CI impedance measurement and electrode illustration.

The measurement setup was designed so that patients only had to connect the audio-processor coil to their implant. It included a Freedom® speech processor with research firmware (ver. 0102E00F02), a clinical programming interface (Pod, Cochlear Ltd.), and the patient's personal computer (Figure 1). Note that it has the same number of elements as for a normal clinical fitting appointment.

### Software

**Figure 2:**
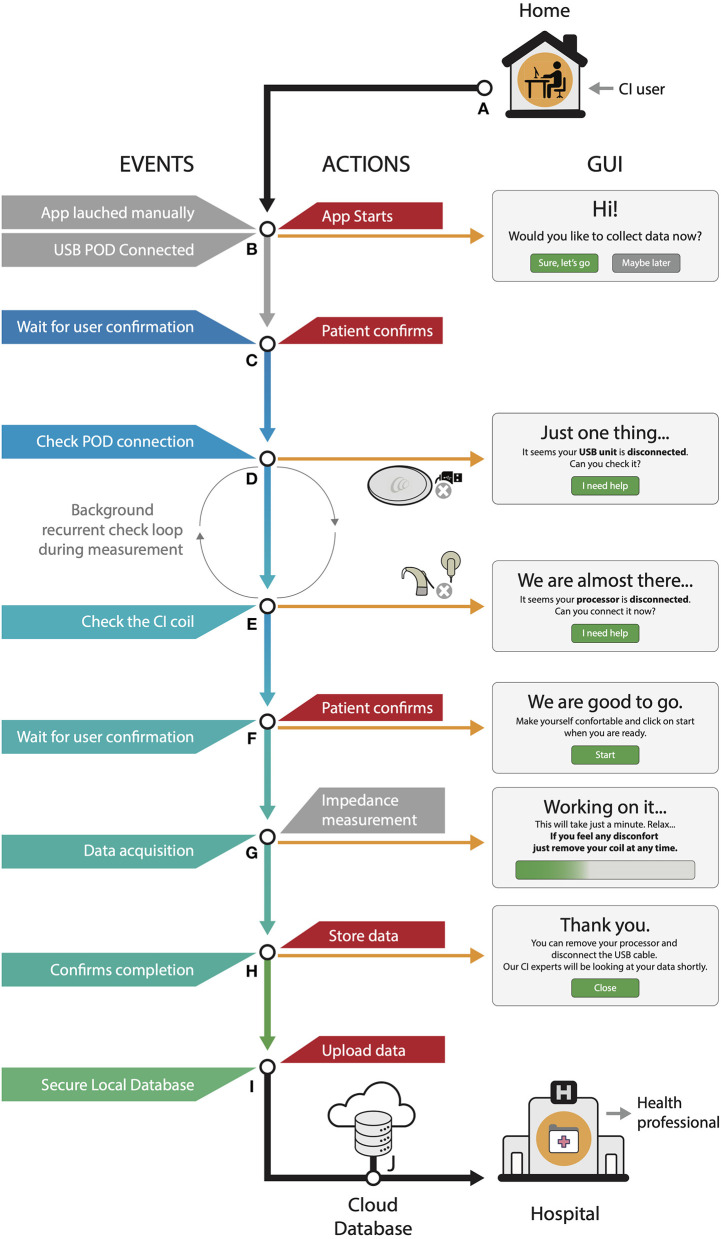
Software workflow **(A–J)** and graphical user interface.

We designed a software that performs CI impedance measurements at home without the intervention of health personnel (Figure 2A). We used the Delphi platform and a dynamic-link library (DLL) provided by Cochlear Ltd. to develop a patient-oriented software that runs under a Windows® operating system and in personal computers. The application was distributed as a single-file installer. Upon installation, it runs in the background automatically detecting the programming Pod connection. The app launches manually or automatically when the Pod is detected. Once running, the main window automatically pops up showing an intuitive and simple graphical user interface (GUI) front end (Figure 2B). The GUI provides the instructions to start the impedance measurements and offers help if it detects incorrect connection to CI (Figures 2D,E). Once an adequate connection is obtained and after user confirmations (Figures 2C,F), it performs impedance measurements (Figure 2G). Each electrode impedance (Z_e_) measurement is assessed by streaming a constant current (I) pulse of 74.21 μA with a phase width of 25 μs. Using the active intracochlear electrode and the extracochlear reference electrode [MP1 coupling mode (8)], voltage (V) at the trailing edge of the pulse is recorded. Finally, the impedance value is calculated through Ohm's law as follows:


(1) $$\begin{array}{r} Z_{e_{n}}\left[\Omega \right]=\frac{V}{I}=\frac{measured\;voltage\;\left[volt\right]}{74.21\cdot 10^{-6}\left[A\right]} \end{array}$$

with *n* being the intracochlear electrode number.

Values are temporarily stored into a secured local database (Figure 2I) by means of the industry-standard SQLite and automatically exported to a web-based secure server (Figure 2J). This process is automated and requires no intervention of the patient. Last, the health professionals with granted access to the cloud database can analyze the CI user data from the hospital.

### Patient Safety

CI impedance measurement is a safe procedure performed as routine in the clinic. It involves stimulating with a low current stimulus which is inaudible for most patients and causes no discomfort (5). Unexpected problems such as connection failure, computer, or software lagging are unable to produce a current level that exceeds defined parameters. This is mainly due to the transmission commands: data packages or tokens carry the information required to perform a required task. In the event of loss of a package, the processor automatically stops all tasks. However, for extra caution or in case of discomfort, the patient was instructed by the investigator and software on how to terminate the procedure immediately by removing the coil (see GUI in Figure 2G).

### Data Security

Both software and connections were developed to ensure maximum protection of the patient's data and anonymity. Information required by the software to register on first use does not include any of the Health Insurance Portability And Accountability Act (HIPAA) identifiers (19). Data transfer to the cloud database only includes deidentified measurements and registration number, which are not associated with the patient's ID. Moreover, the exchanged traffic between the web server and both patient and investigator uses Hypertext Transfer Protocol Secure over transport layer security (HTTPS over TLS). These protocols provide encryption, data integrity, and authentication; thus, reasonable protection is ensured (20).

Patient measurements are temporarily stored in a local encrypted SQLite database (Figure 2I) until data is transmitted to the web server (Figure 2J). Upon transfer to the server, local information is deleted to mitigate risk of local breach. When an internet connection is available, transfer is immediate; otherwise, periodic attempts every 3 min are performed. Impedance acquisition parameters, such as current level and pulse width, are embedded in the software, making its alteration highly improbable.

### CI Subjects

Impedance measurements were conducted after approval of the local Ethics Committee in concordance with international standards for human research. Written informed consent was provided to all participants. A total of 10 CI users were recruited for this study. All patients were implanted at the Hospital Italiano in Buenos Aires, Argentina. A Cochlear Nucleus CI24RE Contour Advance™ electrode array with Freedom or Profile platform (Cochlear Ltd., Australia) was used for all subjects. This CI consists on an array of 22 active electrodes tonotopically arranged inside the cochlea and uses an extracochlear (reference) electrode for MP1 coupling mode (see Figure 1).

The average patient age was 34 (range 1–67). Table 1 shows patient description, including age at implantation, supervisor's age in case of a minor CI user, gender, CI side, and etiology.

**Table 1:** Demographic and general information about participants.

**Subject**	**Age at implantation**	**Supervisor age**	**Gender**	**Implanted ear**	**Etiology**
S1	1	38	F	Right	Preterm—ototoxicity
S2	13	40	M	Right	Ototoxicity
S3	34	–	M	Right	Viral parotitis
S4	16	43	M	Left	Unknown
S5	49	–	F	Left	Otosclerosis
S6	63	–	M	Left	Unknown
S7	41	–	F	Left	Genetic
S8	59	–	F	Left	Unknown
S9	6	30	F	Right	Ototoxicity
S10	67	–	M	Right	Unknown

### Impedance Measurement

To assess CI impedances, the pulse characteristics (i.e., amplitude, phase duration, and interphase gap) were configured according to values used in Custom Sound Suite Software (Cochlear Ltd.) (21). The impedance coupling mode was limited only to Monopolar 1 (MP1), where the circuit is closed using an intra-cochlear and extra-cochlear electrode, where the last operates as the reference for all measures. This external electrode is normally positioned between the skull and the temporal muscle, also referred to as “ball” electrode in Cochlear Ltd. devices. Every time the patient runs the measurement session, a stream of 22 pulses is sent (one for each electrode), and each corresponding voltage telemetry measurement is retrieved. This procedure is performed in accordance with the predefined parameters embedded in the software code. The stream of pulses and recording of each electrode voltage is completed in ~10 s. Note that the entire procedure also includes the connection of the POD and change of audio processor (see Figure 1), which extends the overall time to ~1–2 min.

All subjects were provided with the previously described custom software and measurement hardware. The research team instructed subjects (and/or supervisors) on how to self-perform the measurements at home twice a day—with ~12 h difference—for 30 days before CI activation. A printed brochure on how to connect and measure was also provided as support. A training measurement session was performed under supervision before the patient went home with the equipment. The first appointment (day 0) was measured postoperatively with help and supervision at the hospital, until the activation day (day 1–day 29) subjects measured themselves at home and the last appointment (day 30) was performed at the hospital again.

## Results

A total of 450 measurement sessions were performed, accounting for 75% of the total expected measurements (2 times × 30 days × 10 subjects = 600 measures). From the non-performed measurements (150 measures), only 1 was due to software or hardware issues and the rest correspond to skipped measurement sessions. Subject 7 did not measure for 24 consecutive days, accounting for 48 lost sessions. Although not all subjects were measured twice a day as required, at least one session per day was performed. No adverse events, pain, or discomfort was reported by participants.

**Figure 3:**
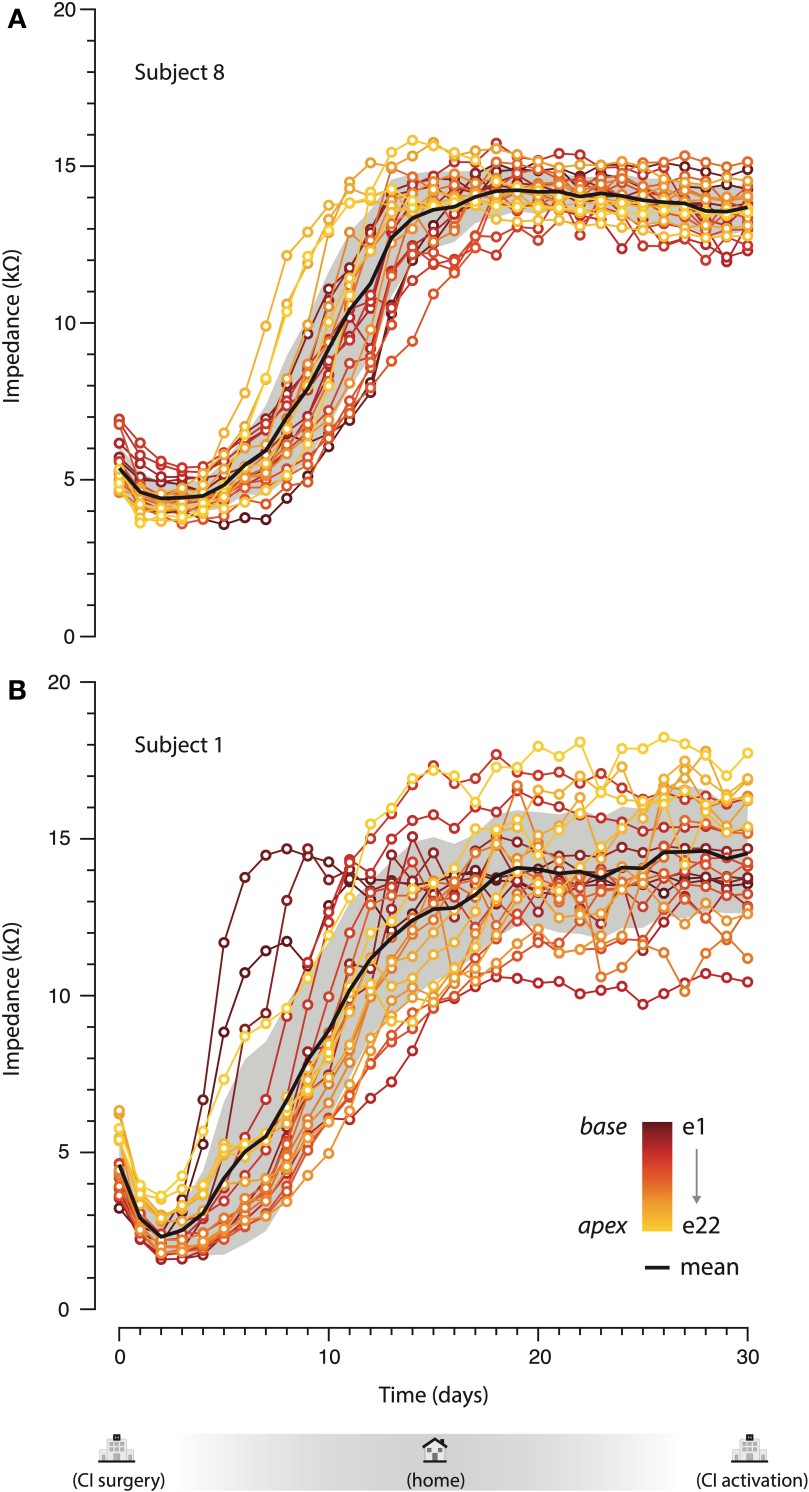
Impedance measurements over time of Subject 8 **(A)** and Subject 1 **(B)**. Colors indicate the electrode number. Black line shows the overall mean over time and the gray patch its standard deviation.

Figure 3 shows two example subjects over time for all electrodes and its overall mean. The average patter of the group is represented by S8 (Figure 3A). All electrodes showed an initial decrease (days 0–3), a continuous growth (4–17 days), and a final stabilization period (days>18). Interestingly, an atypical variation between days 5 and 10 was captured for S1 (Figure 3B), where higher values were observed for the basal electrodes (e1, e2, and e3). Despite the differences across electrodes, they all converge to a more stable value from day 18 approximately to the activation day.

**Figure 4:**
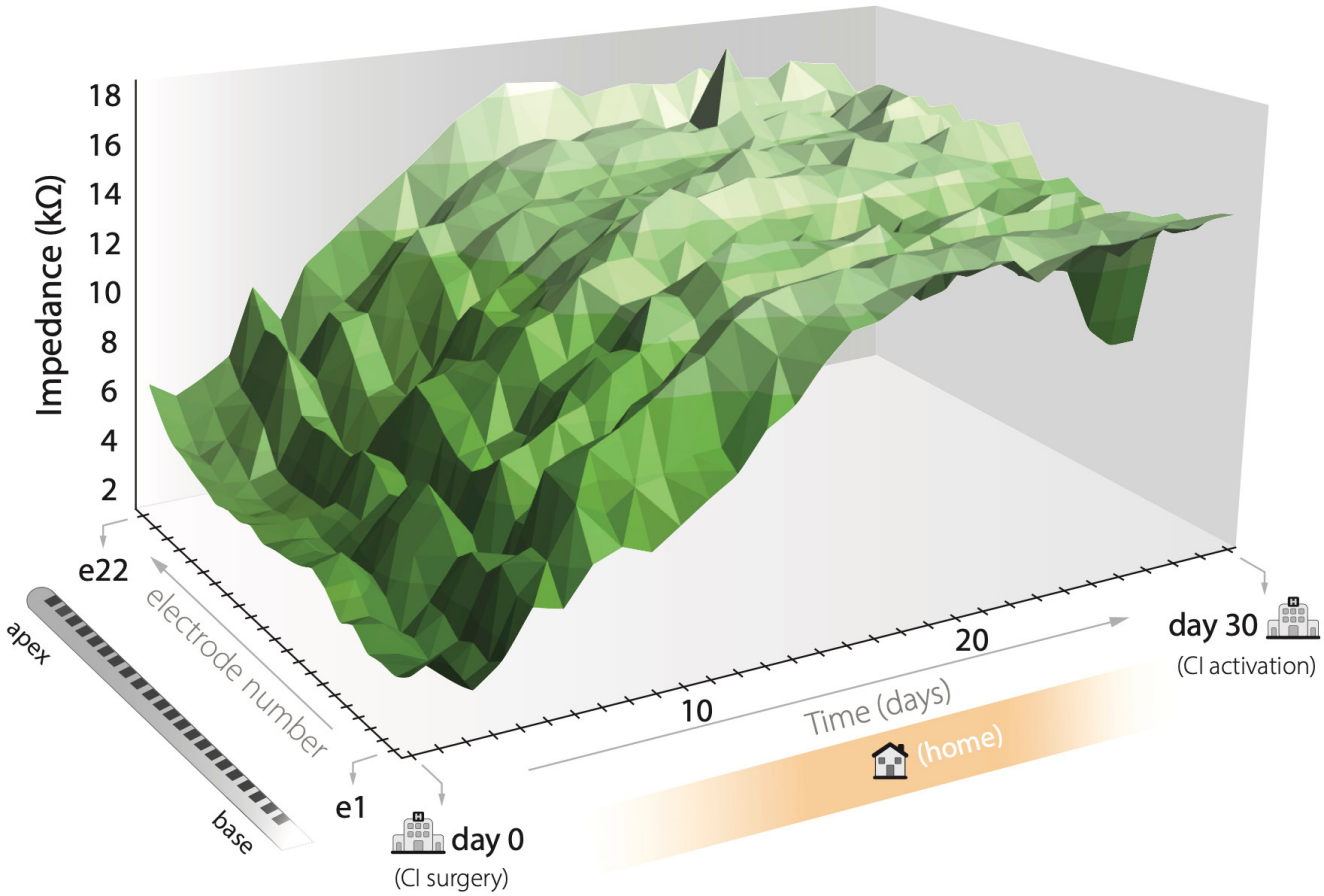
Average impedance value progression pooled for all subjects. First, electrodes are located at the base of the cochlea with higher electrode numbers in the apex. Day 0 was measured postoperatively at the hospital, days 1–29 at home by the patient, and day 30, CI activation, at the hospital again.

To illustrate the overall impedance behavior over time, we computed the average electrode impedance values across subjects over each electrode contact. As shown in Figure 4, average electrode impedance values increased until reaching a plateau at approximately day 15. However, small variations can be observed between electrodes and daily shifts are present. Overall, the group showed impedances of 6.1 kΩ on the surgical day along electrodes and 13.7 kΩ on the activation day.

## Discussion

### A Novel Method

To the best of our knowledge, this is the first report of daily patient remote self-objective measurement in cochlear implants. All CI users (and supervisor) were able to self-perform measurements effectively and in little time. Adherence to measurement was high, allowing precise tracking of clinical impedance evolution on a daily basis.

Given the increasing number of implanted patients and the geographical spread all around the world, the possibility of acquiring remote measurements saves travel costs, time, and physical requirements in clinical care centers. Furthermore, this approach can generate extensive data collection helping to understand overall trends, hidden patterns, unknown correlations, etc.

The presented platform is highly versatile, enabling the integration of other measurements. For example, a more complex measurement of impedance includes polarization impedance and access resistance, which helps to reveal the underlying cochlear pathophysiology mechanism of these changes (7, 22, 23).

In our study, CI users performed their measurement with their personal computer, using specialized research hardware (POD and research CI processor, see methods). However, actual and future connectivity of personal mobile devices (i.e., mobile phones or tablets) allows for streaming of telemetry data, enabling impedance measurements protocols as well as other rehabilitation practices (e.g., audiometry test, speech in noise evaluation, questionnaires). These devices connect wirelessly to the patient audio processors which also can simplify measurements, especially in the pediatric population. More “homemade” measurements in the CI population will substantially improve the CI standard of care, simplifying actual unnecessary procedures and benefiting both the CI user and clinical care centers.

It is important to highlight that an important limitation of this procedure is the requirement of patient collaboration. Most of the lost measurements in our study were due to lack of subject cooperation. Considering that this investigation was carried out during the first month after implantation, where patient expectation on the CI is high, it is likely that this cooperation is further diminished with time. Although we did not assess user's feedback or satisfaction (e.g., via surveys), overall subjects positively agreed with the benefits of “homemade” measures. However, a systematic assessment of user's experience would certainly gain knowledge toward an optimized patient-oriented design. Recently Cochlear Ltd. released a smartphone app to perform remote impedance measurements and other tests in CI users (Cochlear's Remote Check). The benefit of this tool is the portability and wireless connectivity to the CI, potentially increasing the user's engagement. However, one could imagine that future applications with constant background impedance monitoring will rule out any cooperation-related issue and substantially increase the data availability.

Interestingly, the actual epidemiological context due to the COVID-19 pandemic imposed on us the challenge of considering new clinical approaches while practicing social distancing. As we continue to navigate the coronavirus pandemic and its economic consequences, telemedicine approaches like the one presented in this study not only promote the needed social distancing but also help to build the future of the CI standard of care.

### About CI Impedance Daily Monitoring

To the moment, impedances in cochlear implants are a series of isolated values in time measured by audiologists during the fitting process. Daily home monitoring brings a whole new field of opportunities for audiologists, surgeons, and researchers. Impedance shifts may relate to clinical manifestations such as vertigo, Meniere-like symptoms, tinnitus, and loss of residual hearing. Unfortunately, the majority of studies are retrospective; thereby, it is difficult to establish a correlation between the symptoms and impedance variations (7–9). More sophisticated methods, such as the one presented in this paper, may allow rapid diagnosis of the impedance variations and a better correlation with the clinical manifestations. When detecting unusual impedance variations (like the one observed on S1; Figure 3B), automatic alerts could be directed to the CI center for further clinical decision and follow-up. These impedance shifts may be responsive to steroids; thus, detecting them on an early basis may allow prompt treatment and outcome improvement (7, 9, 24). Furthermore, the surgical approach adopted by the surgeon and the electrode insertion itself can cause trauma at the basal turn of the cochlea, which might elicit higher impedances due to its inflammatory process (25, 26).

It is noteworthy that even after impedance stabilization values continue to vary (see Figure 3), which could affect hearing perception even over the course of the same day. Continuous real-time measurement may also improve our results by the development of future auto fitting algorithms and automatic medical referral when values exceed defined parameters.

### Impedance Dynamics Over Time

During the following 2–3 weeks from the surgery, the body's immune response is evidenced by a fibrous tissue encapsulation of the electrode array, which is reflected in a systematic overall increase on the impedance (3, 4, 8, 27–30). Once the CI is activated, the provided electrical current has major implications on the electrode–electrolyte interface (28). Typically, the impedance decreased and then stabilized within the first few months of device use (8, 28, 29, 31–36).

Hu et al. (37) showed the impedance dynamics when activating the CI 1 day after surgery and measuring intraoperatively and postoperatively. This study was performed with the same CI device and shared the first period of measurements as the presented in this paper. Overall, measurements started with a mean 7.9 kΩ intraoperatively and showed an average decrease of 1.9 kΩ at the activation day and a subsequent rise reaching 8.9 kΩ after 8 weeks. Interestingly, the initial impedance drop at the activation day was substantially higher than that observed in our data (mean of 200 Ω; Figure 4). This could be associated with the difference of electrical current provided between studies, since we delivered sub-threshold stimulation which potentially reduced the polarization effect on the inner ear medium. Moreover, Hu et al. reported that 28 days postoperatively the group showed an average of 8.7 kΩ while in our case values reached a mean of 13.6 kΩ. We also argue that this difference could also be due to the interaction of the natural inflammatory process (observed in this study) with the increasing electrical stimulation provided after CI activation.

In conclusion, the method in this paper could be of potential use to better understand the different factors that can play a role on the impedance dynamics over time by offering two main advantages: increased amount of data and measurement simplicity for the CI users and centers.

## Conclusion

This work overviews a flexible and configurable software platform for CI users, which allows self-measures of CI impedance. The outcome enables a remote check of CI status, substantially reducing patients' clinical appointments. All patients performed the measurements in a very short time and without complications. This novel approach can be used to quickly relate a change in the objective measures with a clinical manifestation. Further advances in the method to fully automate measurements are required.

## Data Availability Statement

The original contributions presented in the study are included in the article/supplementary material, further inquiries can be directed to the corresponding author/s.

## Ethics Statement

The studies involving human participants were reviewed and approved by Hospital Italiano, Buenos Aires, Argentina. Written informed consent to participate in this study was provided by the participants' legal guardian/next of kin.

## Author Contributions

FD, MP, and FF designed the methodological approach and collected the data. SA performed the data analysis. FD, MP, FF, and SA wrote the articule. CB supervised the findings and revised final manuscript. All authors contributed to the article and approved the submitted version.

## Conflict of Interest

The authors declare that this study received equipment from Cochlear Ltd. They were not involved in the study design, collection, analysis, interpretation of data, the writing of this article or the decision to submit it for publication.
